# The Importance of Cardiac Magnetic Resonance in the Assessment Risk of Cardiac Arrhythmias in Patients with Arterial Hypertension

**DOI:** 10.3390/jcm13185383

**Published:** 2024-09-11

**Authors:** Andrzej Wysocki, Piotr Macek, Barbara Dziadkowiec-Macek, Małgorzata Poręba, Paweł Gać, Rafał Poręba

**Affiliations:** 1 Centre of Diagnostic Imaging, 4th Military Hospital, 50-981 Wroclaw, Poland; 2 Department of Internal and Occupational Diseases, Hypertension and Clinical Oncology, Wroclaw Medical University, 50-556 Wroclaw, Poland; 3 Department of Paralympic Sports, Wroclaw University of Health and Sport Sciences, 51-617 Wroclaw, Poland; 4 Department of Environmental Health, Occupational Medicine and Epidemiology, Wroclaw Medical University, 50-345 Wroclaw, Poland; 5 Department of Angiology and Internal Medicine, Wroclaw Medical University, 50-556 Wroclaw, Poland

**Keywords:** arrhythmias, arterial hypertension, cardiac magnetic resonance, right ventricular insertion point

## Abstract

**Objectives**: Arterial hypertension (AH) is one of the major risk factors for cardiovascular diseases. An association between untreated AH and arrhythmia is observed. Cardiac magnetic resonance (CMR) assesses myocardial fibrosis by detecting foci of late gadolinium enhancement (LGE). Clinical significance of LGE at the right ventricular insertion point (RVIP) is not fully established. This study aimed to assess the relationship between the presence of LGE at the RVIP determined by CMR and the incidence of arrhythmia in a group suffering from arterial hypertension. **Methods**: The study group consisted of 81 patients with AH (37 men and 44 women, age: 56.7 ± 7.1 years). All subjects underwent CMR and 24 h Holter ECG monitoring. Two subgroups were distinguished in the study group based on the criterion of the presence of LGE at the RVIP in CMR. The RVIP+ subgroup consisted of patients with LGE at the RVIP, while the RVIP− group consisted of patients without LGE at the RVIP. **Results**: The RVIP+ subgroup was characterized by higher maximum and minimum heart rates in 24 h Holter ECG recordings compared to the RVIP− subgroup (*p* < 0.05). The RVIP+ subgroup had a statistically significantly higher number of single premature supraventricular beats, supraventricular tachycardias, and single premature ventricular beats than the RVIP− subgroup (*p* < 0.05). Regression analysis documented that a longer duration of AH (counted from diagnosis) as well as the occurrence of LGE at the RVIP (assessed by CMR) are independent risk factors for arrhythmia (*p* < 0.05). **Conclusions**: Due to the possibility of detecting LGE at the RVIP, CMR may be a useful diagnostic method in estimating the risk of arrhythmias in the group of patients with AH.

## 1. Introduction

Arterial hypertension (AH) is one of the main modifiable cardiovascular risk factors. AH affects around 1.3 billion people aged 30–70 [1,2]. The etiology of arterial hypertension is very complex, with the majority (90–95%) of patients suffering from primary arterial hypertension with a multifactorial gene–environment etiology [3]. The most common complications of arterial hypertension mainly involve the kidney [4], the heart [5,6,7], and retinal vessel lesions [8,9,10]. Simultaneously, arterial hypertension is the most common cause of hypertensive heart disease [5], including left ventricular hypertrophy [11], left atrial enlargement [12], functional mitral regurgitation [13], and neurohormonal changes. Untreated arterial hypertension increases the risk of arrhythmia as well as sudden cardiac death [14,15,16].

Cardiac magnetic resonance (CMR) is a non-invasive cardiac imaging technique to evaluate volume, function, and perform an analysis of myocardial tissue characteristics. CMR is the gold standard for assessing, e.g., the ventricular ejection fraction. The information obtained from CMR allows a comprehensive evaluation of the heart and provides additional information on the etiology of heart failure [17]. CMR allows us to determine the etiology of myocardial damage. The ischemic changes are characterized by rapid myocardial edema, which can be detected in T2-weighted sequences as early as 30 min after the onset of symptoms, as well as late gadolinium enhancement (LGE) with subendocardial to transmural distribution. Non-ischemic cardiomyopathy shows LGE with a mid-wall or subepicardial distribution pattern [18,19]. In patients with hypertrophic cardiomyopathy, two main types of LGE patterns have been distinguished: subepicardial or midmyocardial LGE, which corresponds to descending fibrosis, and right ventricular insertion point (RVIP) LGE, corresponding to interstitial fibrosis and/or myocyte dysfunction [20,21]. The presence of LGE at the RVIP on CMR in patients with other heart diseases, including structural heart diseases, is generally believed to be nonspecific. Additionally, in patients with other structural heart diseases, the RVIP is usually not associated with a worse prognosis [22]. However, the clinical significance of LGE in the RVIP has not been fully established. Grigoratos et al. conducted a retrospective among 2000 patients from which 420 ones with normal CMR images were selected, and in this group LGE was diagnosed in the RVIP in 36 patients. Based on follow-up, they showed that patients with LGE at the RVIP were less likely to have cardiac events than patients without LGE. In contrast, patients with LGE elsewhere (with a non-ischemic pattern) in the myocardium had a worse prognosis than patients with LGE in the RVIP [22].

This study aimed to assess the relationship between the presence of late gadolinium enhancement at the right ventricular insertion point (RVIP) determined by CMR and the occurrence of cardiac arrhythmias in patients with arterial hypertension.

## 2. Materials and Methods

This study is a single-point observational study, conducted between 2019 and 2023. This study included patients of the hypertension department of the University Hospital in Wroclaw, hospitalized to assess the effectiveness of hypertension treatment, assess the organ consequences of arterial hypertension, and possibly optimize the current therapy. The following inclusion criteria were used: age > 18 years, hypertension diagnosed according to the European Society of Hypertension guidelines [1], and willingness to participate in the study. The exclusion criteria were as follows: secondary arterial hypertension, heart failure, coronary artery disease, cardiomyopathy, respiratory insufficiency, renal insufficiency, severe mental disorders, active malignancy, and active inflammation.

This study enrolled 81 patients diagnosed with arterial hypertension. The mean age was 56.7 ± 7.1. Women comprised 54.3% and men 45.7%. A total of 64.2% of the participants were treated with combination therapy. The mean duration of arterial hypertension (counted from diagnosis) was 14.3 ± 4.8 years.

The basic characteristics of the group are shown in Table 1.

This study was conducted based on the principles of the Helsinki Declaration. The study project was positively reviewed by the Bioethics Committee of the Wrocław Medical University. The clinical examination methodology included a medical history, measurement of total cholesterol, fasting glucose, and cardiac magnetic resonance. Blood pressure values were measured using the Korothov method. Total blood cholesterol concentration and fasting blood glucose concentration were determined using standard tests, based on the manufacturer’s instructions.

Twenty-four-hour (from 6:00 to 6:00 the next day) Holter ECG recordings were made with a Lifecard CF 12-channel recorder serial number LIFE-045348/2015 and recording analysis with Sentinel Spacelabs Healthcare Pathfinder SL version 1.7.1.5164 with serial number 8395 (Delmar Reynolds, Hertford, UK). Two experienced cardiologists reviewed the study. ECG recordings were analyzed for quantitative and categorical changes. The variable “sum of categorical changes in 24 h Holter ECG” is a variable that comprehensively summarizes the analysis of the ECG recording in terms of categorical changes. This variable was calculated as follows. Each categorical change that was found in the patient’s ECG recording was assigned 1 point. After analyzing the ECG recording in terms of all assessed categorical variables, the summed points gave the quantitative variable “sum of categorical changes in 24 h Holter ECG”.

Cardiac magnetic resonance (CMR) was performed using one 1.5T Magneton Aera machine (Siemens Healthcare, Forchheim, Germany), according to the same protocol, with a bolus of 0.2 mmol/kg body weight of gadobutrol (Gadovist, Bayer Healthcare, Leverkusen, Germany) administered through the veins of the antecubital fossa. CINE-type SSFP (steady-state free precession sequence), short-tau inversion-recovery (STIR) sequences, late gadolinium enhancement (LGE) sequences, as well as T1 mapping and T2 mapping sequences were performed. LGE imaging was performed using a T1-weighted segmented inversion-recovery pulse sequence, 10 min after administration of the contrast agent. The following LGE sequence parameters were used: slice/gap thickness: 10/0 mm, matrix: 256 × 192, in-plane resolution: 1.4 × 1.4 mm^2^, TR/TE: 650/4.9 ms, flip angle: 30°, inversion time set to null normal myocardium [23]. T1 and T2 mapping sequences were obtained in end-diastole in short-axis orientation in three slices (basal, midventricular, and apical). For myocardial T1 mapping, a MOLLI (modified Look-Locker inversion) recovery acquisition scheme with motion correction (MOCO) was applied. The following T1 mapping sequence parameters were used: slice/gap thickness: 10/0 mm, matrix: 256 × 192, in-plane resolution: 1.4 × 1.4 mm^2^, TR/TE: 305.8/4.9 ms, flip angle: 35°. T2 mapping was performed using a T2-prepared balanced steady-state free precession sequence. The following T2 mapping sequence parameters were used: slice/gap thickness: 10/0 mm, matrix: 256 × 192, in-plane resolution: 1.4 × 1.4 mm^2^, TR/TE: 218.2/1.0 ms, flip angle: 70° [24].

Post-processing evaluation of CMR images was performed using Medis Suite 4.0 software (Medis, Leiden, The Netherlands). CINE sequences were performed in the short axis of the left ventricle and in the long axis in 2-chamber, 3-chamber, and 4-chamber views. In CINE sequences, the dimensions of the cardiac chambers and the functional parameters of the left ventricle were assessed. Images in the short axis and 2-chamber projections in the long axis of the left ventricle were used to estimate the parameters of left ventricular function using the volumetric method. The end-diastolic and end-systolic volumes (EDV and ESV) were calculated as the sum of the left ventricular cavity surface in subsequent layers in images in the short axis multiplied by the thickness of the layer. The stroke volume (SV) was the difference between EDV and ESV. The ejection fraction (EF) was obtained by stroke volume divided by end-diastolic volume. Functional parameters of the left ventricle are presented as body surface area (BSA) indexed values. The left ventricular mass index (LVMI) was calculated too. Foci of left ventricular myocardial edema were assessed in the STIR sequence, and foci of late gadolinium enhancement (foci of myocardial damage) were assessed in the LGE sequence. The myocardium was assessed for the presence of 4 typical types of LGE foci: subendocardial (adjacent to the endocardium but not the epicardium), midwall (not adjacent to the endocardium and the epicardium), subepicardial (adjacent to the epicardium but not the endocardium), and transmural (adjacent to the endocardium and the epicardium). The presence of LGE foci was assessed in 16 segments of the left ventricular myocardium: 6 basal, 6 midventricular, and 4 apical. In addition, the presence of LGE foci in the RVIP was assessed [23]. In T1 and T2 mapping sequences performed before administration of the contrast agent, the native T1 and T2 times of the myocardium were measured. In the T1 mapping sequence performed 20 min after administration of the contrast agent, the post-contrast T1 time (T1 C+) of the myocardium was measured. T1 and T2 relaxation times were analyzed using a dedicated Syngo.via post-processing system (Siemens Healthcare, Forchheim, Germany). The interventricular septum (excluding areas with LGE at the RVIP) was outlined in all three slices (basal, midventricular, and apical) and the averages of all three measurements were obtained [25]. In the assessment of the mapping sequence, the standards estimated for our CMR laboratory were used: up to 1052 ms for T1 time and up to 52 ms for T2 myocardial time.

CMR images analysis was performed consensually by one cardiovascular radiologist with over 10 years of experience in CMR assessment who had passed the ESC EACVI Cardiovascular Magnetic Resonance Exam and achieved national certification in cardiovascular radiology and one cardiologist with over 20 years of experience in cardiac and vascular imaging.

Statistical analysis was performed using the statistical application “Dell Statistica 13.1” (Dell Inc., Round Rock, TX, USA). Quantitative variables were presented in the format of arithmetic means ± standard deviations. Normality of the distributions of variables was verified using the Lilliefors and W–Shapiro–Wilk tests. For quantitative variables meeting the condition of normal distribution, a parametric test, i.e., the *t* test, was used in the comparative analysis. For quantitative variables that did not meet the condition of normal distribution, the hypotheses in the comparative analysis were verified using a nonparametric test, i.e., the Mann–Whitney U test. Categorical variables were presented in the format of numbers/percentages. For categorical variables, the Chi-squared test was used in the comparative analysis. The relationships between variables were assessed in the regression analysis using the backward stepwise multivariate method. The estimation parameters in the regression analysis were obtained using the least squares method. The results were statistically significant at two-sided *p* < 0.05.

## 3. Results

The mean values of the left and right atrial areas were 27.8 ± 7.6 and 21.4 ± 4.3 cm^2^, respectively. The mean left ventricular mass index was 74.3 ± 14.7 g/m^2^. The mean left ventricular ejection fraction was 66.1 ± 6.1%. The late gadolinium enhancement occurred in 33.3% of patients. All LGEs were localized at the right ventricular insertion point. No LGE was demonstrated elsewhere. Myocardial T1 time was 1024.9 ± 8.1 ms, and myocardial T2 time was 42.4 ± 2.7 ms. In all patients, myocardial T1 time and myocardial T2 time were normal.

**Figure 1:**
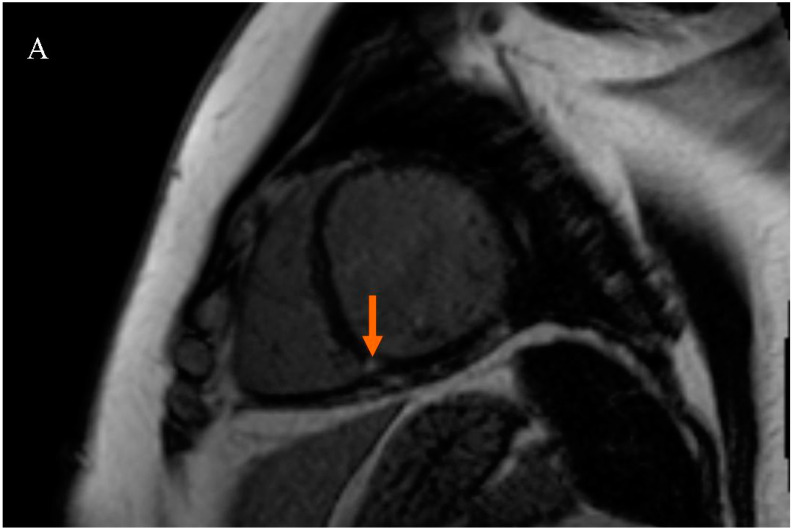

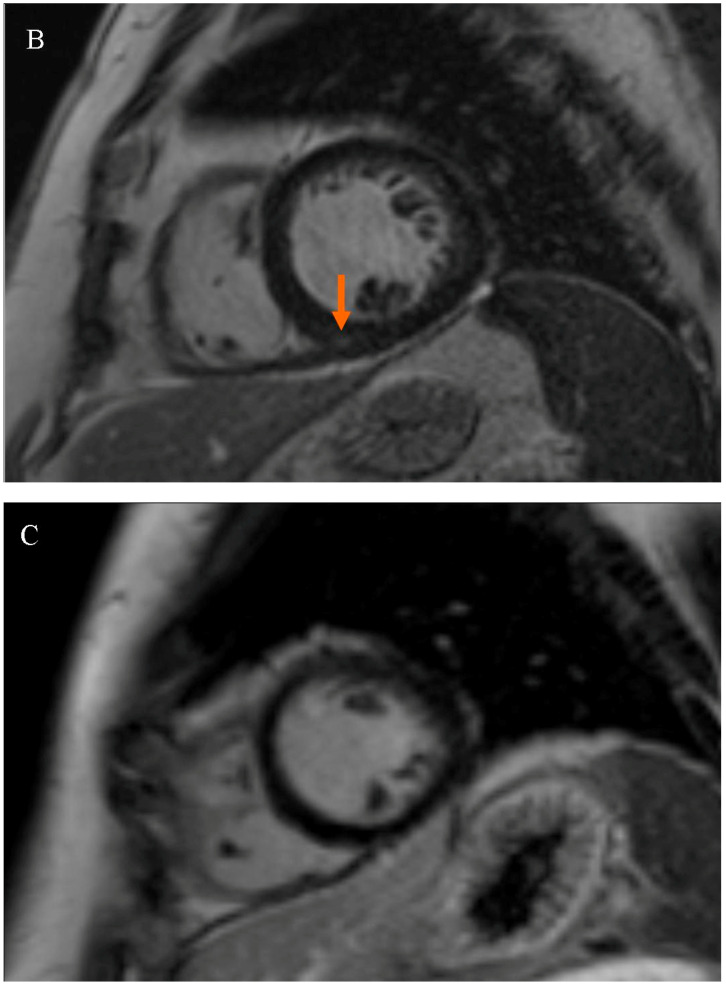
Late gadolinium enhancement (LGE) sequence images in cardiac magnetic resonance (CMR): (**A**) in a female patient with an LGE focus on the right ventricular insertion point (indicated by an orange arrow), (**B**) in a male patient with a subtle LGE focus on the right ventricular insertion point (indicated by an orange arrow), and (**C**) in a patient without LGE foci.

Patients were divided into two subgroups based on the presence of LGE at the RVIP. The first subgroup consisted of subjects with LGE at the right ventricular insertion point (RVIP+ subgroup), while the second subgroup consisted of subjects without LGE at the right ventricular insertion point (RVIP− subgroup), Figure 1.

Details of the parameters analyzed based on CMR are shown in Table 2.

CMR showed that patients in the RVIP+ subgroup had a statistically significantly higher mean left atrial area compared to the RVIP− subgroup (33.0 ± 5.3 vs. 24.4 ± 5.6 cm^2^; *p* < 0.05). Details of CMR parameters in the studied subgroups differing in the occurrence of the RVIP are presented in Table 3.

The Holter ECG showed that patients in the RVIP+ subgroup had a statistically significantly higher mean heart rate compared to the RVIP− subgroup (71.3 ± 6.9 vs. 65.9 ± 6.8 bpm; *p* < 0.05). It was observed that patients with LGE at the RVIP had a significantly higher maximum heart rate of 104.8 ± 13.4 bpm compared to patients without LGE at the RVIP at 98.6 ± 14.1 bpm; *p* < 0.05. Patients in the group with LGE also had significantly more supraventricular premature complex and supraventricular premature complex pairs than the other study participants, 217.2 ± 401.6 vs. 8.1 ± 10.8; *p* < 0.05 and 18.6 ± 45.3 vs. 1.2 ± 1.4; *p* < 0.05, respectively. Details of the other parameters analyzed are presented in Table 4.

Interestingly, patients with LGE at the RVIP were significantly more likely to be diagnosed with atrial fibrillation than patients without LGE at the RVIP, 14.8 vs. 1.8%; *p* < 0.05. Patients in the RVIP+ subgroup were significantly more likely to have tachycardia than participants in the RVIP− subgroup, 18.5 vs. 3.7%, respectively; *p* < 0.05. LGE at the RVIP was not observed to affect the presence of bradycardia, pauses, or atrioventricular blocks. However, patients in the RVIP+ subgroup had significantly more frequent ST-T segment changes than the others, 22.2 vs. 13.0%; *p* < 0.05. Considering the sum of all changes observed on the Holter ECG, patients with LGE in the RVIP had significantly more changes than patients without LGE in the RVIP, 4.2 ± 0.6 vs. 0.9 ± 0.5%; *p* < 0.05. Detailed results are presented in Table 5.

To verify the relationship between the occurrence of LGE in the RVIP and arrhythmias, regression analysis was performed using the “sum of categorical changes in 24 h Holter ECG” variable as the dependent variable. The potential independent variables associated with the sum of categorical changes in the 24 h Holter ECG were the clinical parameters presented in Table 1 (i.e., anthropometric variables, variables characterizing arterial hypertension and treatment of arterial hypertension, variables of lipid and carbohydrate metabolism and smoking) and CMR parameters (variables related to the size and mass of the left ventricle and RVIP). Multivariable, stepwise, backward analysis was used. The assessed model is presented in Table 6.

Regression analysis showed that LGE at the RVIP and a longer duration of arterial hypertension (counted from diagnosis) are independent risk factors for a larger sum of categorical changes in the 24 h Holter ECG.

## 4. Discussion

This study provides additional information on the importance of LGE in the RVIP and its relations with arrhythmias determined by Holter ECG in patients with arterial hypertension. According to the latest guidelines [26], CMR is a technique aiming to recognize cardiomyopathies and various causes of heart failure. Our study showed that patients with arterial hypertension and LGE at the RVIP had a significantly higher average heart rate and maximum heart rate compared to patients without LGE at the RVIP. Furthermore, these patients significantly more often experienced supraventricular premature complexes than patients without LGE. The presence of LGE at the RVIP led to a significantly higher frequency of diagnosed atrial fibrillation in patients compared to those without LGE at the RVIP. Regression analysis demonstrated that LGE at the RVIP and a longer duration of arterial hypertension (counted from diagnosis) were independent risk factors for observed changes in the 24 h Holter ECG.

Arterial hypertension causes endothelial dysfunction, exacerbates the atherosclerotic process, and contributes to the instability of atherosclerotic plaques [27]. Moreover, it leads to left ventricular hypertrophy. All of the abovementioned processes contribute to decreased coronary reserve and increased myocardial oxygen demand, causing myocardial ischemia [28]. Furthermore, arterial hypertension causes harmful effects on the structure of the heart muscle, resulting in left ventricular hypertrophy and excessive growth of fibroblasts and extracellular matrix (fibrosis), death of cardiomyocytes, and heart failure [29]. The intercellular matrix is involved in influencing cardiac cell physiology. Cardiovascular diseases have been proven to affect the remodeling of the matrix and can consequently give rise to impaired cardiac function [30]. Additionally, arterial hypertension is a significant origin in the development of non-rheumatic atrial fibrillation and other supraventricular arrhythmias [31,32]. Hennersdorf et al., based on analyzed studies, showed that the presence of arterial hypertension even without left ventricular hypertrophy significantly increases the risk of ventricular arrhythmias compared to patients with controlled blood pressure. In contrast, left ventricular hypertrophy increases the risk of ventricular tachycardia and mortality fourfold [33].

One of the classic consequences of arterial hypertension is left atrial enlargement. In addition to eliciting left ventricular hypertrophy, dysfunction, and heart failure, hypertension also causes left atrial remodeling that may culminate in atrial contractile dysfunction and atrial fibrillation. The data imply that left atrial remodeling is multifactorial, starts early in hypertension, and is an important contributor to the progression of hypertensive heart disease, including the development of atrial fibrillation and heart failure [12]. Volume and pressure overload is one of the mechanisms of the development of left atrial enlargement in arterial hypertension. Similarly, some researchers believe that the nonspecific symptom of the RVIP is the result of changes in pressure–volume relationships during the cardiac cycle [34]. In our study of patients with hypertension, the subgroup of patients with the RVIP+ symptom was characterized by higher LAA and a more frequent occurrence of supraventricular arrhythmias (including atrial fibrillation) than the RVIP− subgroup. The mechanism explaining this fact may be atrial volume–pressure overload—a common mechanism, as indicated by literature data, for the occurrence of RVIP and left atrial enlargement in arterial hypertension.

Therefore, the common pathophysiology linking the occurrence of the RVIP in CMR, left atrial enlargement, supraventricular arrhythmias, and higher heart rate values may be the volume–pressure overload of the left heart chambers in hypertension. Therefore, it is not surprising that in our study, patients with AH from the RVIP+ subgroup showed higher HR values than patients with AH from the RVIP− subgroup. The above observation could be important in the context of recognizing tachycardia as a potential cardiovascular risk factor. It should be noted, however, that heart rates in the RVIP+ group remained within the normal range. Furthermore, maximum HR obviously depends on what the subject did during the Holter’s 24 h.

CMR is the gold standard for cardiac imaging and has advantages over echocardiography in evaluating and differentiating left ventricular hypertrophy. The finding of left ventricular hypertrophy alone does not establish a clear etiology, which may be related to myocardial fibrosis and abnormal protein deposition. A detailed assessment of the structure of the muscle can be analyzed by CMR [35]. There are various techniques used in CMR. One of them is late gadolinium enhancement, which is a standard evaluation in cardiac imaging. It allows differentiation of ischemic and non-ischemic cardiac damage through characteristic enhancement patterns and is ideal for detecting areas of focal scarring and or fibrosis [36,37]. Other recognized methods include native T1 mapping, which is sensitive to increased tissue water content and is prolonged in cases of inflammation, myocardial edema, and fibrosis and decreased by high iron or lipid content [38,39]. T2 mapping, on the other hand, is used to detect acute inflammation and myocardial edema [40].

Our study has examined whether LGE at the RVIP is significantly associated with arrhythmias. Wang et al. conducted a study showing that the native T1 value of the intraventricular RVIP can play an essential role in the risk stratification of patients with pulmonary hypertension, which may be necessary for deciding the appropriate treatment [41]. Similar observations were indicated in connective tissue disease patients with pulmonary hypertension [42]. Cabanis et al., who based on their studies on sheep and human hearts, showed that the RVIP is a well-organized aggregated cardiomyocyte arrangement. Altering the structure of this site, the presence of LGE may play a role in the formation of cardiac arrhythmias [43].

In contrast to our study, Grigoratos et al. showed that patients with LGE at the RVIP had significantly fewer cardiac events than patients with LGE at another site. In contrast, the Kaplan–Meier curve analysis showed no significant differences between patients with RVIP-LGE and those without LGE at the RVIP. Patients with another LGE had a worse prognosis than patients with RVIP-LGE or no LGE [22]. Similar observations were demonstrated by Yi et al., who showed, based on a study, that LGE found at the RVIP among patients with non-ischemia-related dilated cardiomyopathy did not significantly increase the risk of adverse cardiac events and provided a better prognosis than LV-localized LGE in the same range [44]. On the other hand, Nitsche et al. showed that interstitial proliferation in the anterior RVIP was associated with hemodynamic changes recorded during cardiac catheterization and was independently associated with the prognosis of patients with heart failure [34].

The presence of LGE correlates with the risk of cardiac events in patients with hypertrophic cardiomyopathy. The absence of LGE was associated with a lower risk of events, while the degree of LGE also predicted the development of end-stage heart failure with reduced ejection fraction [45].

Not many studies are available in the literature on the relationship between LGE at the RVIP and cardiac arrhythmias. Available studies show that LGE at the RVIP is not significantly associated with cardiac arrhythmias. It has been shown that the presence of fibrosis at the RVIP can be used in the prognosis of patients with pulmonary hypertension. We, in turn, have shown that patients with LGE at the RVIP and with arterial hypertension had significantly more frequent cardiac events than patients without LGE.

Our results may also be important in the ongoing discussion on heart remodeling in athletes. As Allwood et al. point out, the development of myocardial fibrosis in athletes appears to be a multifactorial process, with genetics, hormones, the exercise dose, and an adverse cardiovascular risk profile playing key roles. Myocardial fibrosis is not a benign finding and warrants a comprehensive evaluation and follow-up regarding potential cardiac disease [46]. Małek et al. documented that in their study group, 73.3% of athletes fulfilled the volumetric criteria for dilated cardiomyopathy or arrhythmogenic right ventricular cardiomyopathy. Non-ischemic, small-volume LGE was found in 27% of athletes and in 10% of controls. It was localized at the RVIP or in the septum or inferolateral wall. Athletes with LGE at the RVIP had a higher right ventricular end-diastolic volume index in comparison to athletes without LGE (*p* < 0.05), which suggests its relation to volume overload [47]. In this context, the relationship we have shown between the presence of LGE at the RVIP and arrhythmias may be a potential link in the pathogenesis of cardiac arrhythmias in athletes.

The limitations of our study were the relatively small number of study participants and the lack of patients in the study group with other types of LGE. Undoubtedly, the issue requires further study to demonstrate the clinical implications of LGE at the RVIP.

## 5. Conclusions

Cardiac magnetic resonance by identifying late gadolinium enhancement at the right ventricular insertion point may be a proper diagnostic method in assessing the risk of cardiac arrhythmias in patients with arterial hypertension.

## Figures and Tables

**Table 1:** Clinical characteristics and characteristics of treatment of arterial hypertension in the whole study group.

Age [years] ^1^	56.7 ± 7.1
BMI [kg/m^2^] ^1^	26.9 ± 3.0
Sex ^2^	
Men	37/45.7
Women	44/54.3
Grades of arterial hypertension according to ESH/ECS 2	
Mild	31/38.3
Moderate	48/59.2
Severe	2/2.5
Treatment of arterial hypertension ^2^	81/100.0
Monotherapy	29/35.8
Combination Therapy	52/64.2
Drugs in the treatment of arterial hypertension ^2^	81/100.0
ACE inhibitors	48/59.2
β-blockers	39/48.1
Diuretics	17/21.0
Calcium channel blockers	29/35.8
Angiotensin receptor blockers	11/13.6
Duration of arterial hypertension [years] ^1^	14.3 ± 4.8
Systolic blood pressure [mmHg] ^1^	138.4 ± 16.9
Diastolic blood pressure [mmHg] ^1^	83.7 ± 8.3
Type 2 of diabetes ^2^	14/17.3
Fasting glucose [mg/dL] ^1^	123.6 ± 37.2
Hypercholesterolemia ^2^	44/54.3
Total cholesterol [mg/dL] ^1^	247.3 ± 48.4
Smoking ^2^	22/27.2
Cigarette years ^1^	307.2 ± 168.1

^1^ Values represent means +/− standard deviations; ^2^ values represent numbers/percentages; ACE—angiotensin-converting enzyme inhibitors; BMI—body mass index; ESC—European Society of Cardiology; ESH—European Society of Hypertension.

**Table 2:** Cardiac magnetic resonance parameters in the study group.

Left atrial area in 4-chamber projection (LAA) [cm^2^] ^1^	27.8 ± 7.6
Right atrial area in 4-chamber projection (RAA) [cm^2^] ^1^	21.4 ± 4.3
Left ventricular end-diastolic diameter (LVEDD) [mm] ^1^	58.7 ± 10.6
Left ventricular end-systolic diameter (LVESD) [mm] ^1^	34.7 ± 11.6
Interventricular septal end-diastolic wall thickness (IVS-EDWT) [mm] ^1^	10.9 ± 1.3
Posterior wall end-diastolic thickness (PW-EDWT) [mm] ^1^	9.3 ± 1.7
Left ventricular mass index (LVMI) [g/m^2^] ^1^	74.3 ± 14.7
Left ventricular end-diastolic volume index (LVEDVI) [mL/m^2^] ^1^	83.4 ± 21.5
Left ventricular end-systolic volume index (LVESVI) [mL/m^2^] ^1^	34.7 ± 13.0
Left ventricular stroke volume index (LVSVI) [mL/m^2^] ^1^	48.7 ± 9.7
Left ventricular ejection fraction (LVEF) [%] ^1^	66.1 ± 6.1
Left ventricular edema ^2^	0/0.0
The ratio of myocardial intensity to skeletal muscle intensity (T2 ratio) ^1^	1.6 ± 0.3
Late gadolinium enhancement (LGE) ^2^	27/33.3
Late gadolinium enhancement (LGE) at the right ventricular insertion point (RVIP) ^2^	27/33.3
Late gadolinium enhancement (LGE) in other locations of the myocardium ^2^	0/0.0
Myocardium T1 time [ms] ^1^	1024.9 ± 8.1
Myocardium T2 time [ms] ^1^	42.4 ± 2.7
Myocardium post-contrast T1 time (T1 C+) [ms] ^1^	443.0 ± 19.6
Pericardial effusion ^2^	0/0.0

^1^ Values represent means +/− standard deviations; ^2^ values represent numbers/percentages.

**Table 3:** Cardiac magnetic resonance parameters in the study subgroups: A—subgroup with late gadolinium enhancement at the right ventricular insertion point (RVIP+); B—subgroup without any late gadolinium enhancement at the right ventricular insertion point (RVIP−).

	RVIP+ (*n* = 27)	RVIP− (*n* = 54)	*p*
Left atrial area in 4-chamber projection (LAA) [cm^2^] ^1^	33.0 ± 5.3	24.4 ± 5.6	<0.05
Right atrial area in 4-chamber projection (RAA) [cm^2^] ^1^	20.5 ± 5.3	21.0 ± 3.4	ns
Left ventricular end-diastolic diameter (LVEDD) [mm] ^1^	60.2 ± 11.9	58.4 ± 9.8	ns
Left ventricular end-systolic diameter (LVESD) [mm] ^1^	35.3 ± 8.7	36.9 ± 11.4	ns
Interventricular septal end-diastolic wall thickness (IVS-EDWT) [mm] ^1^	10.2 ± 1.3	9.9 ± 1.4	ns
Posterior wall end-diastolic thickness (PW-EDWT) [mm] ^1^	9.7 ± 1.6	9.2 ± 2.0	ns
Left ventricular mass index (LVMI) [g/m^2^] ^1^	79.5 ± 17.4	78.8 ± 16.9	ns
Left ventricular end-diastolic volume index (LVEDVI) [mL/m^2^] ^1^	86.4 ± 16.8	82.8 ± 22.3	ns
Left ventricular end-systolic volume index (LVESVI) [mL/m^2^] ^1^	37.2 ± 14.2	35.8 ± 12.9	ns
Left ventricular stroke volume index (LVSVI) [mL/m^2^] ^1^	47.7 ± 9.8	50.2 ± 11.6	ns
Left ventricular ejection fraction (LVEF) [%] ^1^	64.4 ± 6.4	66.7 ± 5.3	ns
Left ventricular edema ^2^	0/0.0	0/0.0	ns
The ratio of myocardial intensity to skeletal muscle intensity (T2 ratio) ^1^	1.5 ± 0.3	1.6 ± 0.2	ns
Myocardium T1 time [ms] ^1^	1024.3 ± 7.6	1025.3 ± 8.5	ns
Myocardium T2 time [ms] ^1^	42.1 ± 2.9	42.6 ± 2.6	ns
Myocardium post-contrast T1 time (T1 C+) [ms] ^1^	439.7 ± 21.4	446.2 ± 18.5	ns
Pericardial effusion ^2^	0/0.0	0/0.0	ns

^1^ Values represent means +/− standard deviations; ^2^ values represent numbers/percentages; ns—non-statistically significant.

**Table 4:** Quantitative parameters of 24 h Holter ECG monitoring in the study subgroups: A—subgroup with late gadolinium enhancement at the right ventricular insertion point (RVIP+); B—subgroup without any late gadolinium enhancement at the right ventricular insertion point (RVIP−).

	RVIP+ (*n* = 27)	RVIP− (*n* = 54)	*p*
HR mean [bpm] ^1^	71.3 ± 6.9	65.9 ± 6.8	<0.05
HR min [bpm] ^1^	56.6 ± 6.4	54.8 ± 7.0	ns
HR max [bpm] ^1^	104.8 ± 13.4	98.6 ± 14.1	<0.05
SVPC ^1^	217.2 ± 401.6	8.1 ± 10.8	<0.05
SVPC pairs ^1^	18.6 ± 45.3	1.2 ± 1.4	<0.05
SVT ^1^	1.4 ± 2.7	0.2 ± 0.1	ns
VPC ^1^	104.6 ± 271.6	67.4 ± 110.6	ns
VPC pairs ^1^	0.6 ± 3.1	0.0 ± 0.0	ns
bigeminy ^1^	0.2 ± 0.8	0.0 ± 0.0	ns
trigeminy ^1^	0.4 ± 0.7	0.2 ± 0.1	ns
sinus tachycardia ^1^	0.2 ± 0.6	0.4 ± 0.9	ns
sinus bradycardia ^1^	0.7 ± 1.1	0.0 ± 0.00	ns
pauses > 2.5 s ^1^	1.1 ± 2.0	0.0 ± 0.00	ns

^1^ Values represent the number of this type of episode/event in the 24 h ECG (in format means +/− standard deviations); HR—heart rate; ns—non-statistically significant; SVPC—supraventricular premature complex; SVT—supraventricular tachycardia; VPC—ventricular premature complex.

**Table 5:** Categorical parameters of 24 h Holter ECG monitoring in the study subgroups: A—subgroup with late gadolinium enhancement at the right ventricular insertion point (RVIP+); B—subgroup without any late gadolinium enhancement at the right ventricular insertion point (RVIP−).

	RVIP+ (*n* = 27)	RVIP− (*n* = 54)	*p*
sinus rhythm ^1^	25/92.6	54/100.0	ns
escape rhythm ^1^	2/7.4	0/0.0	ns
AF ^1^	4/14.8	1/1.8	<0.05
VT ^1^	1/3.7	1/1.8	ns
sinus tachycardia ^1^	5/18.5	2/3.7	<0.05
sinus bradycardia ^1^	1/3.7	0/0.0	ns
pauses > 2.5 s ^1^	2/7.4	0/0.0	ns
I-degree atrioventricular block ^1^	8/29.6	11/20.4	ns
Wenckebach’s II-degree atrioventricular block ^1^	2/7.4	1/1.8	ns
Mobitz’s II-degree atrioventricular block ^1^	0/0.0	0/0.0	ns
III-degree atrioventricular block ^1^	1/3.7	0/0.0	ns
ST-T changes ^1^	6/22.2	7/13.0	<0.05
∑ categorical changes ^2^	4.2 ± 0.6	0.9 ± 0.5	<0.05

^1^ Values represent the number/percentage of patients in whom this type of episode/event was observed in the 24 h ECG; ^2^ values represent means +/− standard deviations; AF—atrial fibrillation; ns—non-statistically significant; ST-T changes—significant ST depressions, negative T waves, significant ST elevations or pathological Q waves; VT—ventricular tachycardia; ∑ categorical changes—total number of points in the categorical assessment of the ECG recording in each patient, where each detected categorical abnormality of the ECG recording was assigned 1 point.

**Table 6:** Results of regression analysis in the study group: risk factors for higher ∑ categorical changes in 24 h Holter ECG monitoring.

Model for: ∑ Categorical Changes in 24 h Holter ECG Monitoring			
	Intercept	LGE at RVIP #	Duration of arterial hypertension (years)
Regression coefficient	0.584	2.628	0.143
SEM of Rc	0.211	1.175	0.059
*p*	<0.05	<0.05	<0.05
statistical power of estimation	*p* < 0.05, SEM of estimation 0.089, corrected R^2^ 0.577		

# Dichotomous variable, where 0—no; 1—yes; LGE—late gadolinium enhancement; RVIP—right ventricular insertion point; SEM—standard error of mean; ∑ categorical changes—total number of points in the categorical assessment of the ECG recording in each patient, where each detected categorical abnormality of the ECG recording (yes/no) was assigned 1 point.

## Data Availability

The data presented in this study are available on request from the corresponding author. The data are not publicly available due to privacy or ethical restrictions.
